# Applying healthcare failure mode and effect analysis to enhance patient-controlled analgesia in acute post anesthesia pain management

**DOI:** 10.3389/fmed.2025.1663936

**Published:** 2025-10-29

**Authors:** Shaoru Chen, Hongmei Zhang, Hui Zhi, Jie Wang

**Affiliations:** ^1^Department of Anesthesia and Perioperative Medicine, Henan Provincial People’s Hospital, Zhengzhou, China; ^2^Henan Provincial Key Medicine Laboratory of Nursing, Zhengzhou, China; ^3^Zhengzhou University People’s Hospital, Zhengzhou, China; ^4^Henan University People’s Hospital, Zhengzhou, China; ^5^Department of Nursing, Henan Provincial People’s Hospital, Zhengzhou, China

**Keywords:** patient-controlled analgesia, postoperative pain management, HFMEA, acute pain, PACU

## Abstract

**Purpose:**

This study aimed to evaluate the application of Healthcare Failure Mode and Effect Analysis (HFMEA) to optimize the patient-controlled analgesia management process for patients experiencing acute pain after general anesthesia.

**Methods:**

In this retrospective study, the experimental group included 475 patients who underwent general anesthesia between July and December 2024, whereas the control group included 503 patients between January and June 2024. The experimental group received an HFMEA-optimized analgesia management process, whereas the control group received the standard nursing protocol. Patients’ pain scores, post-anesthesia care unit (PACU) stay length, and risk priority number (RPN) values were compared before and after HFMEA implementation.

**Results:**

Following implementation, RPN values decreased from high to low risk, pain scores dropped significantly, and PACU stay was shortened (*p* < 0.05).

**Conclusion:**

Implementation of an HFMEA-optimized analgesia process for patients with acute post-general anesthesia pain improves pain control and speeds recovery.

## 1 Introduction

Patient-controlled analgesia (PCA) is a therapeutic method in which clinicians preprogram analgesic doses based on the patient’s physiologic condition and pain intensity, enabling the patient to self-administer medication for pain relief. PCA reduces perioperative pain, thereby suppressing the stress and inflammatory responses induced by surgical trauma (1, 2). PCA is easy to use, provides effective analgesia, and accelerates patient recuperation after surgery. It is a crucial tool in postoperative pain management (3). The Chinese Society of Anesthesiology’s 2024 “Expert consensus on the Clinical Application of Patient-Controlled Analgesia” noted that PCA should adhere to the “on-demand analgesia” principle and be tailored to patient-specific needs across clinical contexts and time points. The efficacy and safety of PCA are compromised by several factors, including insufficient clinical awareness among medical staff, ambiguous role delineation, ineffective doctor-patient communication, and the absence of standardized management and quality assessment protocols (3).

The Joint Commission on Accreditation of Healthcare Organizations (JCAHO) formally recognizes healthcare failure mode and effect analysis (HFMEA). This methodology prospectively optimizes and verifies risk procedures in medical processes, thereby reducing adverse events within the medical quality management model (4, 5). The basic process consists of six steps: identifying the theme, assembling the team, developing the process, analyzing the risks, creating an action plan, and assessing the outcome. This procedure involves conducting a prospective quantitative analysis of potential process failures, identifying their underlying causes, and implementing targeted improvements to systematically prevent these failures and reduce the frequency of medical risks (6). It plays a role in pain management (7), emergency nursing (8), post-anesthesia care unit (PACU) quality management (9, 10), and operating room nursing (11). However, reports on the application of the HFMEA model to patient-controlled analgesia for acute pain following general anesthesia remain scarce. As patients often have a limited understanding of PCA, clinicians must provide comprehensive assessments, clear instructions, and diligent supervision to ensure effective postoperative pain relief (3).

In this study, the HFMEA model was used to evaluate the failure modes and influencing factors across each link of the PCA management plan for patients with acute pain after general anesthesia, analyze the key links, formulate an optimized PCA management plan, and accelerate the perioperative surgical recovery of patients.

## 2 Materials and methods

### 2.1 Inclusion and exclusion criteria

The experimental group comprised 475 patients who received general anesthesia in the PACU between July and December 2024, whereas the control group included 503 patients who received general anesthesia in the same setting between January and June 2024. This study had a retrospective cohort design. This study adhered to the provisions of the Helsinki Declaration, safeguarded the privacy of the participants.

The inclusion criteria were as follows: (i) intubated patients transferred to the PACU after general anesthesia, (ii) American Society of Anesthesiologists (ASA) grade III-IV, (iii) Numerical Rating Scale (NRS) > 3, and (iv) no adverse events before or during the operation.

The exclusion criteria were as follows: (i) severe cardiopulmonary or cerebral disease not transferred to the PACU, and (ii) preoperative assessment of dementia, cognitive impairment, or inability to communicate effectively. There were no significant differences in age, sex, department, or ASA grade between the two groups (*p* > 0.05), as shown in Table 1.

**Table 1:** Comparison of general data of patients with acute pain after general anesthesia between the two groups.

Item		Control group (*n* = 503)	Intervention group (*n* = 475)	t	*p*
Gender	Male	279 (0.55)	272 (0.57)	0.320	0.571
	Female	224 (0.45)	203 (0.43)		
Age (years)		48.14 ± 18.90	46.53 ± 19.52	1.313	0.189
ASA	III	62 (0.12)	68 (0.14)	0.839	0.360
	IV	441 (0.88)	407 (0.86)		

### 2.2 Healthcare failure mode and effect analysis (HFMEA)

The first step was to determine the research theme: “Effect evaluation of optimizing PCA management process for patients with acute pain after general anesthesia based on HFMEA”.

#### 2.2.1 Step 2: set up the HFMEA team

One anesthesiologist, one head nurse, five nursing team leaders in the recovery room, and one pain management nurse were among the physicians and nurses who collaborated to form the HFMEA team. All had a bachelor’s degree or higher, and there were six intermediate titles, one senior title, and one associate senior title. Following the team’s formation, members completed a systematic study of the HFMEA model and the research issues through both online and offline theoretical teaching techniques. After completing the test, they were able to understand the HFMEA research procedures and safety measures before beginning the actual operation process. The team held sporadic quality control meetings, conducted frequent business conversations, and set up an online communication group.

#### 2.2.2 Step 3: draw the flow chart of patient-controlled analgesia management for patients with acute pain after general anesthesia

Through field tracking, brainstorming, literature analysis, and clinical practice experience, the HFMEA team members examined the postoperative PCA process for patients under general anesthesia in the PACU. The sub-process under the main process was improved, and steps requiring optimization were identified based on the timing and workflow. Five steps were identified and included in the flow chart of PCA management for patients experiencing acute pain following general anesthesia: patients were transferred to the PACU, anesthesiologists were consulted, patients’ conditions were monitored during the recovery period, patients with a Steward score ≥ 4 were moved out of the PACU per the doctor’s orders, and the patients were transferred to the ward.

#### 2.2.3 Step 4: identify the potential failure modes, conduct risk analysis, and calculate the risk priority index

From the sub-processes, potential failure modes, failure causes, and potential effects, the HFMEA project team used brainstorming, literature analysis, and expert group meetings to examine the six processes in managing a PCA pump for patients experiencing acute pain following general anesthesia, supplemented by clinical experience. The failure risk priority number (RPN) was determined, which is essential when applying the HFMEA paradigm for continuous quality management. The determinants of RPN are Occurrence (O) and severity (S) (12). Failure severity was graded into four categories using the clinical medical risk severity grading standard: extremely serious, severe, moderate, and mild. These categories received scores of four, three, two, and one points, respectively, with higher scores indicating greater severity (Table 2). Each of the four grades—frequent, occasional, infrequent, and rare— was given 4, 3, 2, and 1 points, respectively, in accordance with the Australian Clinical Medical Risk Likelihood Assessment Grading standard model; higher scores indicated greater failure probability (Table 3). With a total score ranging from 1 to 16, the RPN = S × O. A higher RPN score indicated a higher risk value, indicating a latent hazard of the process (Table 4). To identify key failure modes, decision tree analysis was used to examine the failure modes with high RPN scores (RPN ≥ 8) and failure modes with high damage severity (RPN < 8) (4, 13) (Table 5).

**Table 2:** Scoring criteria for failure mode severity (S).

Level	NRS score	Score
Extremely serious	NRS:9 ~ 10	4
Severe	NRS:7 ~ 8	3
Moderate	NRS:4 ~ 6	2
Mild	NRS:1 ~ 3	1

**Table 3:** Occurrence (O) scoring criteria.

Level	Standard of scoring	Score
Frequent	It often occurs or occurs for a short period of time	4
Occasional	May occur (several times within 1 to 2 years)	3
Uncommon	May occur (have occurred within 2 to 5 years)	2
Remote	Hardly ever (once in 5 to 30 years)	1

**Table 4:** Hazard scoring matrix.

Severity occurrence	Extremely serious (4)	Severe (3)	Moderate (2)	Mild (1)
Frequent (4)	16	12	8	4
Occasional (3)	12	9	6	3
Uncommon (2)	8	6	4	2
Remote (1)	4	3	2	1

**Table 5:** Hazard scores and decision tree analysis.

Process steps	Failure mode	Potential causes of failure	Hazard score			Decision tree analysis			
			S	O	RPN	Single point weakness	Existing control measure	Detectable	Proceed
Patients under general anesthesia were transferred to PACU	Patient-controlled analgesia pump information was not checked before patients left the operating room	The verification process of analgesic pump needs to be standardized	3	3	9	→	N	N	Y
Handover with the anesthesiologist	The delivery of analgesia pump was not standardized (such as no label, wrong writing of label information, inconsistent parameter Settings and label information)	Some doctors and nurses were not familiar with the parameter setting and adjustment of analgesic pump	3	3	9	→	N	N	Y
The condition was observed during the recovery period	The pain was not treated in time after the patient woke up	Some doctors and nurses used pain assessment tools incorrectly	4	3	12	→	N	N	Y
Steward ≥4 was transferred out of PACU following medical advice	The patient-controlled analgesia pump was not checked again by the anesthesia nurse before the patient left the PACU	Some doctors and nurses were not familiar with the verification process of analgesic pump	3	3	9	→	N	N	Y
Transfer to ward	Patient-controlled analgesia training and health education were not in place	1. Ward nurses were not familiar with the alarm handling of patient-controlled analgesia pump	4	2	8	→	N	N	Y
		2. Incorrect timing and usage of patient-controlled keys							

#### 2.2.4 Step 5: develop and implement improvement measures

**Figure 1:**
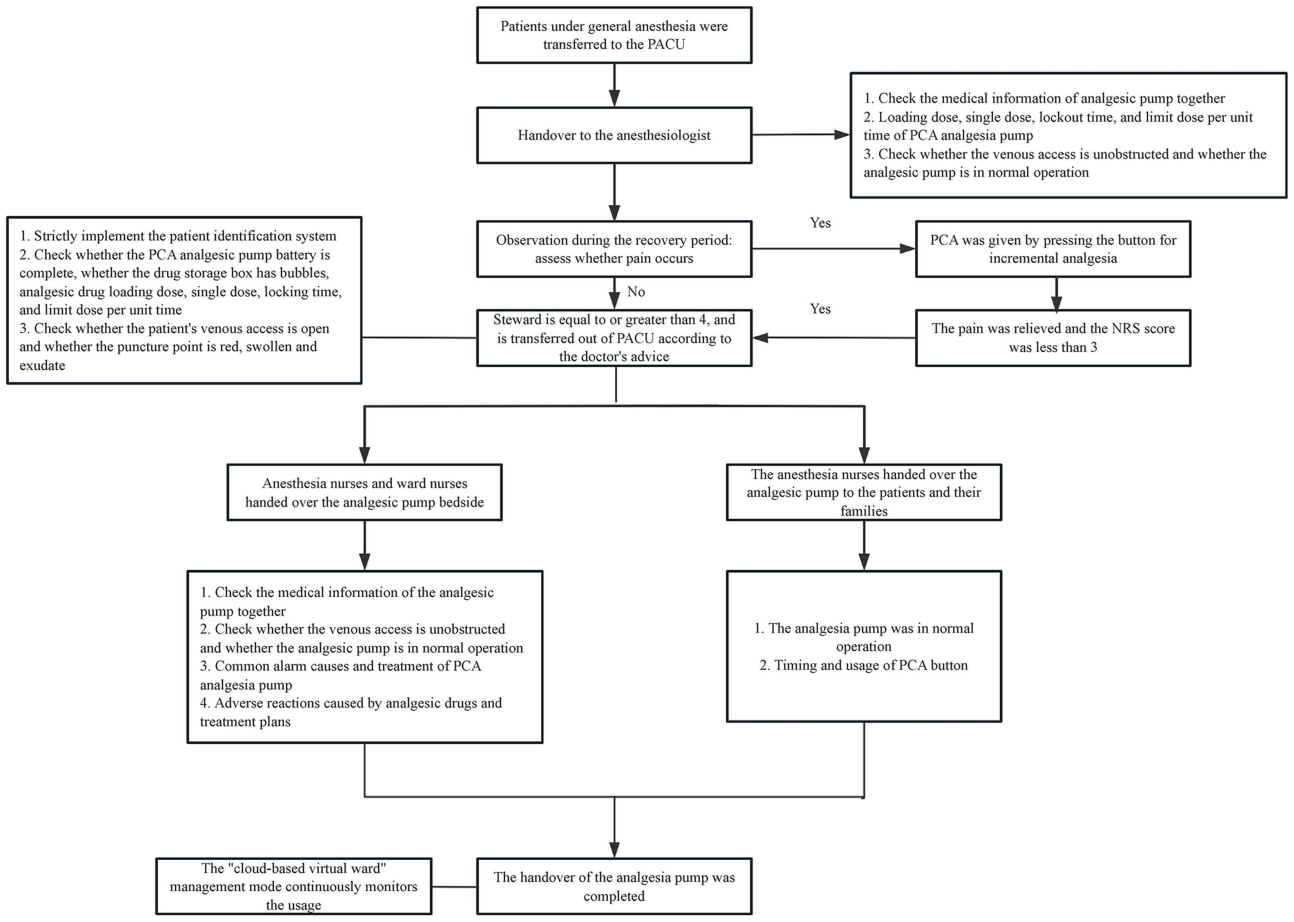
Flow chart of patient-controlled analgesia management for patients with acute pain after general anesthesia. (i) Power alarm: this alerts nurses to connect the power or replace the battery when the power is off or the battery display is too low. (ii) The alarm is activated and the obstruction’s cause is addressed when the infusion pipeline or the catheter’s front end becomes blocked or broken. (iii) Air alarm When air bubbles form in the infusion pipeline, they should be released to avoid the bubbles getting into the body and raising concerns. (iv) The analgesic pump is promptly removed and placed in the “analgesic pump recycling box” in the treatment room when the alarm at the conclusion of the infusion is triggered.

After multidisciplinary discussions, the HFMEA project team identified six major failure modes through decision tree analysis, examined and categorized the possible causes of failure, and developed improvement strategies. Optimization details are provided in Table 6. To assist the medical staff in the PACU in accurately implementing pertinent measures, a clear and refined verification flowchart for PCA in patients with acute pain following general anesthesia was constructed based on the optimized content as illustrated in Figure 1. To optimize the entire process, the team established task divisions, coordinated the work of physicians and nurses, delivered scheduled training and supervision, promptly communicated and coordinated existing details online, and held group quality control meetings.

**Table 6:** List of improvement actions.

Process	Measures for improvement
Patients under general anesthesia were transferred to PACU	Patient information, connection and usage parameters of patient-controlled analgesia pump were checked before transport
Handover with the anesthesiologist	1. Medical staff should be trained on the use of analgesic pump through on-site demonstration and lecture. 2. Formulate the management standard of anesthesia analgesia pump.
The condition was observed during the recovery period	1. Training patients on pain scoring, and correctly using the corresponding scoring tools for different groups
Steward ≥4 was transferred out of PACU following medical advice	1. The anesthesia nurse should implement the patient identification system again before the patient leaves the PACU 2. The anesthesia nurse checked whether the analgesic pump had bubbles, whether it was blocked, the machine had sufficient power, and the parameters were set correctly 3. Develop a flow chart of patient-controlled analgesia management for patients with acute pain
Transfer to ward	1. Carry out analgesic pump use courses for ward nurses 2. Record the use of the analgesic pump, and paste the two-dimensional code on the analgesic pump bottle

#### 2.2.5 Step 6: observation index

(i) Evaluation of the RPN value for PCA pump management before and after HFMEA deployment in patients experiencing acute pain following general anesthesia. (ii) Patient pain rating. (iii) Time spent in the PACU before and after the improvement measures were implemented.

### 2.3 Statistical analysis

Data entry was performed using excel, and statistical analyses were performed using SPSS version 25.0. The mean ± standard deviation was used to describe measurement data that fit a normal distribution, whereas the frequency and constituent ratios were used to describe count data. Continuous normal variables were tested using the two independent sample *t* test, whereas categorical variables were tested using the *χ*^2^ test. Statistical significance was set at *p* < 0.05.

## 3 Results

### 3.1 Comparison of RPN values of PCA in patients with acute pain after general anesthesia before and after HFMEA

The project team reassessed the risk value of the PCA pump management process six months after HFMEA management was introduced into the PCA management process for patients experiencing acute pain following general anesthesia. As indicated in Table 7, the risk value was significantly lower than before the adoption of HFMEA (*p* < 0.05).

**Table 7:** Comparison of RPN values before and after HFMEA implementation (x̅ ± *s*).

Group	Patient-controlled analgesia pump information was not checked before patients left the operating room	The handover of analgesia pump was not standardized	The pain was not treated in time after the patient woke up	The patient-controlled analgesia pump was not checked again by the anesthesia nurse before the patient left the PACU	Patient-controlled analgesia training and health education were not in place	(x̅ ± *s*)
Before HFMEA	9	9	12	9	8	9.40 ± 1.52
After HFMEA	6	4	6	4	6	5.20 ± 1.10
*t*						5.020
*P*						<0.05

### 3.2 The pain scores and PACU length of stay were compared before and after HFMEA

The project team reassessed the pain incidence and PACU retention time of the PCA pump management process six months after HFMEA management was introduced into the PCA management process for patients experiencing acute pain following general anesthesia. As indicated in Table 8, the risk value was significantly lower than before the adoption of HFMEA (*p* < 0.05).

**Table 8:** Comparison of pain incidence and PACU retention time before and after HFMEA (x̅ ± *s*).

Group	*n*	NRS		PACU stay time (h)
		Before treatment	After treatment	
Before HFMEA	503	7.14 ± 1.71	1.04 ± 0.21	2.83 ± 0.37
After HFMEA	475	4.36 ± 0.64	1.08 ± 2.09	2.01 ± 0.21
*t*		33.953	−2.411	42.544
*p*		<0.05	0.016	<0.05

## 4 Discussion

### 4.1 The application of HFMEA model in risk assessment of PCA management process for patients with acute pain after general anesthesia can optimize the management process and reduce the management risk

The HFMEA management approach enhances nursing quality and aids in the development of medical management processes. To achieve standardized management and improvement of the evaluation index, HFMEA can prospectively evaluate the project process, effectively highlight weaknesses in the medical care process management, precisely identify failure modes and causes, create action plans, and clearly reflect the evaluation index of the project’s improvement effect (14). The five primary processes of PCA management for patients experiencing acute pain following general anesthesia were identified in this study: transferring patients under general anesthesia to the PACU, handoff to anesthesiologists, monitoring the patients’ condition during the recovery phase, transferring patients with a Steward score ≥ 4 out of the PACU in accordance with the doctor’s orders, and transferring to the ward. The potential risk factors were analyzed and refined to create focused improvement strategies and streamline procedures. The findings demonstrated that the risk values of the failure modes in every stage of the PCA management process for patients experiencing acute pain following general anesthesia significantly decreased following the implementation of HFMEA management, and the differences were statistically significant (*p* < 0.05). This demonstrates that the HFMEA-based PCA management procedure for patients experiencing acute pain following general anesthesia can lower the risk of multiple factors during the procedure, enhance overall process safety, and show viability and efficacy. To prevent postoperative patient-controlled anesthetic oversedation events, Cronrath et al. (15) used the HFMEA model to manage complex processes. The predetermined HFMEA goal—a 50% decrease in oversedation events—was accomplished after a year. In the study, Sun et al. (11) verified that the use of the HFMEA model in sputum specimen management can enhance specimen quality and the rate of positive detection. Zhao’s research, which utilized HFMEA approach to manage postoperative diabetes insipidus in pediatric neurosurgery, helped optimize the management process, alleviate postoperative symptoms of diabetes insipidus, and improve the prognosis (16).

### 4.2 The application of HFMEA model to optimize the PCA management process of patients with acute pain after general anesthesia can improve the effect of pain management, reduce the incidence of pain, and the length of PACU stay

The HFMEA is a crucial instrument for diagnosing health systems and a potent tool for enhancing professional health learners’ knowledge and capacity for quality improvement (17). In this study, pain scores and PACU length of stay were significantly lower after the implementation of HFMEA, and the differences were statistically significant (*p* < 0.05) after the HFMEA model was used to optimize the PCA management process for patients with acute pain following general anesthesia. Healthcare organizations should conduct prospective risk analyses in high-risk processes at least once a year according to JCAHO. The requirements for high-risk processes are as follows: an internal unusual event report suggests that the event is frequent or of high severity; a sentinel event shows that the event poses a risk to patient safety; an external source indicates that the event is frequent or of high severity; or a new system or procedure is required (18). An examination of the literature revealed that acute pain following general anesthesia is common and, if left untreated, can adversely affect patients (19). Thus, the PCA management procedure for patients experiencing acute pain following general anesthesia was optimized, and the frequency of associated adverse events decreased in accordance with the expert consensus on PCA management (2). Sun et al. (4) used the HFMEA model. To improve medication safety management, a multidisciplinary team evaluated the risk of intrathecal morphine pump use and prioritized steps to lower this risk. This allowed nursing managers to shift safety events associated with intrathecal morphine pump administration from negative treatment to positive prevention prior to the event. It guarantees nursing safety and represents an ongoing enhancement of nursing quality.

In conclusion, as a quality management methodology, HFMEA uses prospective analysis to identify emerging issues, enable preventive action before issues arise, and prevent adverse events by addressing root causes. One limitation of this study is its retrospective design relying on historical data, which may affect the accuracy of outcome analyses. Prospective cohort studies will be conducted to optimize future designs. This study examined causes of PCA failure in the anesthesia and perioperative medicine departments and aimed to improve the PCA procedure to greatly enhance patient pain management and reduce the duration of PACU stay. As a result, applying HFMEA to the PCA management process for acute pain following general anesthesia can proactively control failure modes and guarantee patient safety, supporting broader clinical adoption.

## Data Availability

The original contributions presented in the study are included in the article/supplementary material, further inquiries can be directed to the corresponding author.
